# Evaluating the feasibility of a decision aid to promote shared decision making among young adults with first-episode psychosis: protocol for a pilot study

**DOI:** 10.1186/s40814-020-00757-0

**Published:** 2021-01-11

**Authors:** Yaara Zisman-Ilani, Irene Hurford, Andrea Bowen, Mark Salzer, Elizabeth C. Thomas

**Affiliations:** 1 Department of Social and Behavioral Science, College of Public Health, Temple University, Philadelphia, PA USA; 2 Psychosis Education, Assessment, Care and Empowerment (PEACE), Horizon House, Philadelphia, PA USA; 3 Department of Psychiatry, Perelman School of Medicine, University of Pennsylvania, Philadelphia, PA USA

**Keywords:** Shared decision making, Decision aid, Psychosis, First Episode Psychosis, Schizophrenia, Emerging adults

## Abstract

**Background:**

Young adults ages 18 to 25 with first episode psychosis (FEP) have an increased risk of discontinuation antipsychotic medications and psychiatric service disengagement that lead to symptom exacerbation and deterioration. We seek to (1) examine the feasibility, usability, and potential impact of a Shared Decision Making (SDM) Antipsychotic Medication Decision Aid (DA) on decision-making, adherence to the decision made, and service engagement among young adults with FEP and (2) understand the role of additional patient-level factors on SDM.

**Methods:**

A randomized controlled trial is being conducted in a coordinated specialty care community program for FEP in an urban setting. Eligible patients are randomly assigned to receive an intervention, the Antipsychotic Medication Decision Aid, or treatment as usual. Patients receive their assigned intervention before their medication appointment with the psychiatrist and complete four interviews: before the appointment (T0), after the appointment (T1), and at 3- and 6-month follow-ups (T2 and T3). The study staff and participating psychiatrists are not blinded to the intervention. The data are de-identified to maintain blinding during the analysis process. The primary aims are feasibility of intervention delivery and research procedures and preliminary impact of the intervention on SDM-related outcomes, medication adherence, and service engagement. As a secondary aim, we will explore the contribution of personality and motivation variables, clinical relationships, cognitive functioning, and mental-health-related stigma to SDM. If the sample size permits, we plan to conduct parametric tests such as independent-samples *t* tests at T1 to compare differences in SDM, adherence, and engagement scales. In the case of a small sample size, we will use non-parametric tests and descriptive statistics.

**Discussion:**

This protocol outlines the methodology for a feasibility pilot comparing the effect of a novel SDM Antipsychotic Medication encounter DA with treatment as usual on SDM, medication adherence, and service engagement in FEP care. SDM is endorsed as a framework for use in FEP and antipsychotic pharmacotherapy, but its impact on adherence and health outcomes is unclear. Understanding the potential contribution of an SDM Antipsychotic Medication DA compared with usual care in psychosis pharmacotherapy is critical. The study will help answer several key questions new to SDM research, including the contribution of personality and clinical relationships to SDM in mental health and psychosis in particular. The study will serve to gather feasibility data to inform future studies and scale-up.

**Trial registration:**

Ethics approval was obtained through Temple University’s institutional review board (IRB) and the City of Philadelphia’s Department of Public Health IRB. The study has been retrospectively registered with ClinicalTrials.gov as NCT04373590 on 29 April 2020. https://clinicaltrials.gov/ct2/show/NCT04373590?term=NCT04373590&draw=2&rank=1

## Background

Psychosis is a pervasive worldwide condition, with schizophrenia being the most prevalent form of psychotic disorder [1]. Approximately one in 150 individuals will be diagnosed with a psychotic disorder at some point during their lifetime [2]. The rates of first episode psychosis (FEP) peak between the ages of 15 and 29 at 126 per 100,000 and young adults ages 18 to 25 have an increased risk of developing recurrent psychotic episodes that lead to a diagnosis of schizophrenia [3, 4]. Prescribing antipsychotic medications is a standard frontline clinical practice for FEP [5, 6]. Yet, discontinuation and nonadherence rates with antipsychotic medications are very high [7–12].

Engaging in shared decision making (SDM) has been recently recognized as an integral strategy for helping patients with serious mental illnesses choose the best treatment for them, increase adherence to their treatment decision, and improve mental health outcomes [13–18]. Decision aids (DA), or decision support tools, are the most common type of SDM interventions that help patients and clinicians make informed, values-consistent treatment decisions by describing and comparing relevant treatment options [19]. In a recent systematic review of SDM for individuals with serious mental illnesses [20], we identified only five studies about engagement of young adults with psychosis in FEP programs, while SDM has been used as a working or conceptual framework but not as a concrete intervention [21–24]. We also identified one study about clinical decision-making (not an SDM) [25], and one study about preparation for SDM, where the intervention was provided by peers before the patient–clinician encounter and discussion but did not address a specific decision or medical condition [26]. These findings suggest that the literature on SDM for young adults with FEP is scarce and that no SDM intervention exists for use as part of medication management appointments.

Antipsychotic pharmacotherapy and decision-making may be particularly complicated during the period of young adulthood due to potential negative impacts on lifestyle and body image. Further, young adults face a number of barriers to their active participation in medication-related decision-making [27]. The primary purpose of this study is to conduct a randomized controlled pilot trial to evaluate the feasibility, usability, and potential effectiveness of an antipsychotic medicaitons DA [28] for antipsychotic medication management on SDM and adherence over time in a sample of young adults with FEP. The secondary purpose is to fill a gap in SDM in mental health research that neglects patient-level psychological concepts that are known to affect clinical relationships, care, and outcomes such as therapeutic alliance, attachment style, and cognitive performance [29–34]. In the present study, we will explore, for the first time, how such patient-level factors are associated with SDM.

## Methods

This clinical trial protocol follows the Standard Protocol Items: Recommendations for Interventional Trials (SPIRIT) 2013 Statement and guidelines [35, 36] and the Consolidated Standards of Reporting Trials (CONSORT) guidelines.

### Study design

The present study uses a longitudinal randomized controlled pilot trial to compare the feasibility and potential effect of a high-quality DA for antipsychotic medications management [28] with treatment as usual and will examine the impact on SDM, medication adherence, and service engagement. In this study, we focus on medication management visits. In the SDM group, the tested intervention was used, whereas in the treatment-as-usual group, no intervention was used and the psychiatrist discussed antipsychotic pharmacotherapy as usual. The study also examines an exploratory hypothesis; how attachment style, cognitive functioning, and therapeutic alliance are associated with SDM outcomes and DA potential effectiveness.

### Specific aims

Aim 1: To evaluate the feasibility and usability of the DA in a coordinated specialty care program for FEP.

Aim 2: To evaluate the potential effectiveness of the DA with treatment as usual on SDM, adherence to treatment decisions, and service engagement at the conclusion of an appointment and over time.

**Hypothesis 2.1:** Patients who receive DA will report greater knowledge about antipsychotic medication, decision self-efficacy, and SDM; positive decision attitudes; and lower decisional conflict scores than those who receive treatment as usual.

**Hypothesis 2.2:** Patients who receive DA will have greater adherence to their treatment decision and greater service engagement over time than those who receive treatment as usual.

Aim 3: To identify patient-level and intervention-level factors associated with the DA. These factors include (1) patient characteristics (e.g., age, gender, education level, and diagnosis), (2) personality and motivation factors (i.e., attachment style, apathy), (3) clinical relationships (i.e., working alliance, trust), (4) cognition (i.e., Cognition in Schizophrenia, insight), (5) self-stigma, and (6) intervention compliance (e.g., whether patients reviewed the DA in the appointment and amount of time spent reviewing the DA).

### Conceptual framework

This study was based on the conceptual framework of SDM as outlined in the work of Charles et al. [37, 38], Montori et al. [39], and Elwyn et al. [40, 41] that views SDM as an approach for enabling continuous improvement in clinical decision-making, healthcare delivery, and patient outcomes. The framework recognizes the fundamental necessity of patient–clinician reciprocity and partnership in the decision-making task and healthcare delivery. The framework also relies on emerging insights related to decision-making in mental health, highlighting the importance of personality, clinical relationships, and cognitive functioning [14, 16, 33, 42]. The SDM DA intervention [28] chosen for this study addresses the key elements of the conceptual framework, helps psychiatrists convey clinical evidence to patients in ways that they can access and understand, and helps patients with FEP communicate concerns and preferences regarding their antipsychotic treatment in a structured manner. In this way, the SDM DA will ensure high-quality antipsychotic medication choice that is evidence-based and patient-centered [43].

### Participants, intervention, and outcomes

#### Setting and participants

Patients and psychiatrists are being recruited from a coordinated specialty care program for FEP in an urban setting on the East Coast of the United States (U.S.). Coordinated specialty care programs are considered the gold standard of FEP care in the U.S. and are built on the positive findings of the Recovery After an Initial Schizophrenia Episode (RAISE) project, funded by the National Institute of Mental Health (NIMH) [44–46]. The coordinated specialty care program was selected because of its access to an ample number of potential patients with FEP ages 18 to 25 and its accessible community location, and because medications are offered as one of the treatment options.

Current coordinated specialty care patients are screened to determine eligibility by a research assistant (RA). Screening takes place in person at the coordinated specialty care program or by telephone using a screening questionnaire with study criteria questions.

The eligibility criteria for patients are as follows:

- Age 18 to 25 years
- Experiencing early psychosis, defined as psychosis lasting 18 months or less between the time when threshold symptom criteria were reached (as determined by the admitting coordinated specialty care program assessor) and the date of coordinated specialty care program enrollment
- Planning to attend a medication appointment with a participating coordinated specialty care psychiatrist
- Ability to speak and understand English
- Ability to provide informed consent as assessed by research staff using procedures discussed by Carpenter et al. [47], including a demonstrated understanding and recall of study procedures, rather than passive consent, and allowance of repetition of study procedures until there is understanding and recall.

Patients with the following will be ineligible:

- Having a legal guardian
- Having identified co-occurring dementia, delirium, or intellectual disability that will likely affect their ability to provide informed consent or participate in the data collection procedures.

#### Intervention

The chosen intervention is an SDM DA developed by the first author (YZI), published and fully described elsewhere [28, 48] for use during the psychiatric consultation to help patients and clinicians discuss relevant treatment options pertaining to antipsychotic medications, including not taking or discontinuing medication. The DA targets a major problem in psychosis care: lack of patient-clinician communciaiton, medication nonadherence and discontinuation [8]. Many people who experience psychosis stop or adjust their medication and dosage without sharing this information with their clinicians or close family [49, 50], which presumably results in worsening of symptoms, disruption of the continuity of care, re-hospitalization, and high mortality rates, a phenomenon known as the “revolving door” [51, 52]. The intervention aims to facilitate an SDM discussion and a “laying of the cards on the table,” including a discussion about the option of stopping antipsychotic medications. Its format is a one-page table with rows containing frequently asked questions by patients about their treatment options and the benefits, risks, and implications of differing decisions. The columns display the treatment options available for the treatment decision in question: continuing, adjusting, or discontinuing antipsychotic medications. The DA has an International Patient Decision Aid Standards (IPDAS) rating by the Ottawa inventory of decision aids [53] and is publicly available along with a supporting evidence document [28].

#### Sample size

**Fig. 1:**
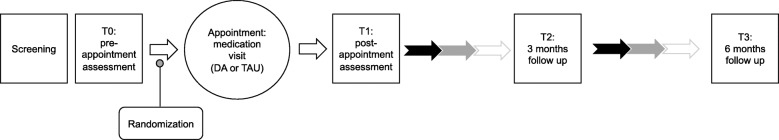
Flow of study intervention and assessments

We planned to recruit patients enrolled in the coordinated specialty care program for baseline T0 (Fig. 1). The sample size was calculated based on an estimate of the site’s enrollment rate. Given that this study’s purposes were to assess feasibility of intervention delivery and research procedures and to collect preliminary data about the intervention potential effectiveness and related patient- and intervention-level factors, the sample size was based on pragmatics rather than on power and is consistent with recommendations for pilot studies [54].

#### Outcomes

Our primary outcomes are SDM, adherence a treatment decision that is collaboratively made, service engagement, and feasibility and usability of delivery. SDM is a complex concept with several components [55, 56]. In this study, we focused on the main components of SDM: knowledge about antipsychotic medications, decision self-efficacy, decision attitudes, decisional conflict, and SDM process and patient satisfaction at a clinical encounter. The following self-report scales were used to measure SDM: a 9-item antipsychotic medications knowledge scale adapted from O’Connor’s knowledge scale [57], the 11-item Decision Self-Efficacy (DSE) scale [58], the 10-item Decision Attitude Scale (DAS) [59], the 15-item Decisional Conflict Scale (DCS) [60], and the 3-item collaboRATE [61]. Medication adherence was measured using the 8-item Brief Adherence Rating Scale (BARS) [62], and service engagement was measured using both the 66-item Service Use and Resource Form for Monthly Items (SURF-M) [63] and the 14-item Service Engagement Scale (SES) [64]. Feasibility and usability is assessed by documenting recruitment procedures (screening and enrollment), randomization (proportion of eligible screens who enroll and received the intervention), treatment-specific retention rates and reasons for dropout, and an SDM implementation report to evaluate the DA usability [65].

Secondary outcomes include SDM-related personality and motivation variables, clinical relationships, cognitive functioning, and mental health-related stigma. The 36-item Experiences in Close Relationships-Revised (ECR-R) Questionnaire [66] and the 18-item Marin Apathy Evaluation Scale [67] are well-validated scales used to measure personality- and motivation-related variables. The 36-item Working Alliance Inventory (WAI) [68] and the 11-item Trust in the Medical Profession Scale [69] are well-validated self-report scales used to measure clinical relationships. The Brief Assessment of Cognition in Schizophrenia (BACS) [70] includes seven sections to assess aspects of cognition such as verbal memory and attention that are found to be most impaired and most strongly correlated with functional outcome in patients with schizophrenia [71]. The 8-item Birchwood Insight Scale [72] was used to assess insight into a mental illness, defined as the ability to recognize one's own mental illness [73]. Finally, the 10-item Internalized Stigma of Mental Illness (ISMI) Scale [74] was used to assess the internalization of public stigma by people with mental illness. Internalized or self-stigma is associated with poor mental health and is a prominent barrier to accessing treatment. Table 1 shows which predictors and outcomes were administered at each timepoint.

**Table 1:** Predictors and outcomes collected at different timepoints

Outcome level, construct, and measure	Baseline (t_**0**_)	Post appointment (t_**1**_)	3-month follow-up (t_**2**_)	6-month follow-up (t_**3**_)
Primary outcomes				
SDM				
Antipsychotic medications knowledge scale	x	x		
Decision Self-Efficacy (DSE) scale		x		
Decision Attitude Scale (DAS)		x		
Decisional Conflict Scale (DCS)		x		
collaboRATE		x		
SDM implementation report		x		
Adherence				
Brief Adherence Rating Scale (BARS)	x	x	x	x
Service Use and Resource Form for Monthly Items (SURF-M)	x	x	x	x
Service Engagement Scale (SES)	x			
Secondary outcomes				
Personality and motivation				
Experiences in Close Relationships-Revised (ECR-R) Questionnaire	x			
Marin Apathy Evaluation Scale	x			
Clinical relationships				
Working Alliance Inventory (WAI)	x			
Trust in the Medical Profession Scale	x			
Cognitive functioning				
Brief Assessment of Cognition in Schizophrenia (BACS)	x			
Birchwood Insight Scale	x			
Self-stigma				
Internalized Stigma of Mental Illness (ISMI) Scale–Brief Version	x			

Information about demographics and clinical characteristics was collected at baseline (T_0_) and included self-report data such as diagnosis, age, gender, education, and race. Because FEP is a minimal condition for enrollment in the collaborating coordinated specialty care program, all participants in the program and in the study have an FEP diagnosis.

#### Delivery of intervention and assessments

The study activities included screening, recruitment and intervention, and survey delivery. The sequence of activities within the coordinated specialty care program flow is illustrated in Fig. 1:

- **Screening and pre-appointment interview (T0):** A trained research assistant (RA) screens new patients from the coordinated specialty care program. Patients are invited to participate in the study, sign an informed consent form, and participate in a first pre-appointment, baseline interview (Fig. 1, Table 1). Because the interview takes about 90 min, it is usually not conducted on the day of a medication appointment. In any case, the pre-appointment interview is conducted before participants see the psychiatrist to discuss their medications.
- **Randomization:** At the end of the T_0_ interview, the RA randomizes patients to the SDM DA intervention or the treatment-as-usual condition.
- **Intervention/treatment-as-usual delivery:** The same psychiatrist introduces the DA for patients assigned to the intervention condition or does not introduce it when patients are assigned to treatment as usual. The psychiatrist receives a notification from the RA on the day of the appointment if the current patient is a study participant and whether they are assigned to the intervention or treatment-as-usual condition. The psychiatrist completes the implementation report after seeing each participant.
- **Post-appointment interview at T1:** After the appointment, the RA collects the implementation report and conducts the second interview that usually occurs on the same day of the appointment or a few days after. Both the pre- and the post-appointment interviews take place in person at the coordinated specialty care program.
- **Three-month follow-up at T2:** Participants are interviewed in person at the coordinated specialty care program or by telephone 3 months after their post-appointment interviews.
- **Six-month follow-up at T3:** Participants are interviewed in person at the coordinated specialty care program or by telephone 6 months after their post- appointment interviews.

#### Recruitment strategies, status, and trial dates

**Fig. 2:**
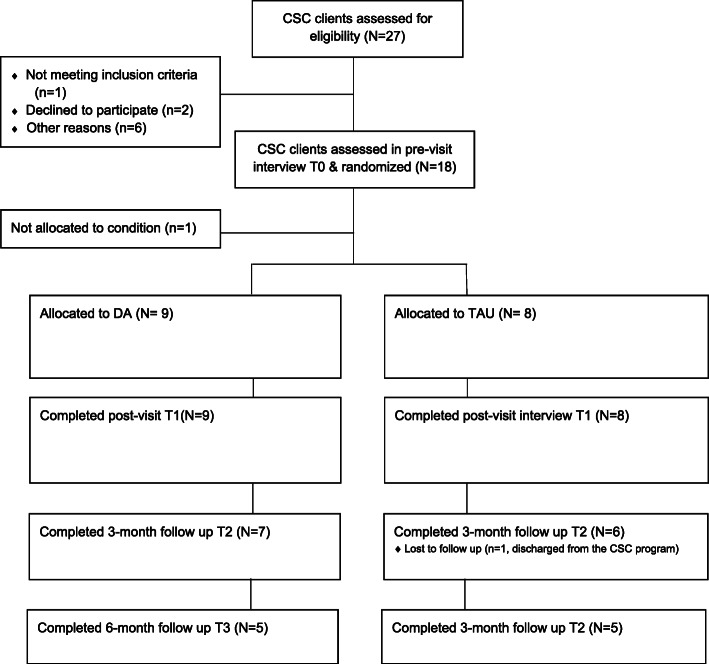
Consolidated Standards of Reporting Trials flow diagram estimating patient screening, enrollment, and response rate. DA, decision aid; TAU, treatment as usual; T_0_, pre-appointment; T_1_, post-appointment; T_2_, 3-month follow-up; T_3_, 6-month follow-up

Figure 2 is the CONSORT flow diagram and includes estimates of screening, enrollment, and response rates. Emerging adults referred to the coordinated specialty care program are recruited to participate using flyers and site presentations. Interested individuals are instructed to contact research staff via telephone. In addition, staff at the coordinated specialty care program are provided information about the study for potentially eligible individuals. The RA follows up with those interested in participation for screening but has no direct access to participants’ identifying information until these individuals have expressed interest in the study. The RA determines eligibility in person at the coordinated specialty care program or by telephone by using a screening questionnaire with study criteria questions. In-person screenings are conducted at the coordinated specialty care program in a private room. The RA fully informs eligible individuals about study procedures. In addition, the RA conducts an assessment to evaluate individuals’ capacity to provide informed consent using a screener developed for the study. Individuals determined to be capable of providing informed consent are invited to attend the pre-appointment research interview. At the beginning of the interview, the RA provides an overview of the study and obtains written informed consent prior to implementing any study procedures. Patient enrollment began on February 2019.

### Randomization and blinding

Randomization occurs at the client level, where patients are randomized to SDM DA or to treatment as usual after enrolling in the study and before their allocation to the study conditions. Randomization is accomplished using an HIPAA-compliant, Internet-based randomization service (studyrandomizer.com) using permuted blocks of five. The RA, who is blind to the random allocation sequence, enters the patient into the randomization service after completion of the pre-appointment interview and notifies them of the condition to which they are assigned.

Patients and the participating psychiatrist are not blinded to the condition assigned to them; however, they are not given any explicit information or training about the intervention. The psychiatrist was provided the DA and received information about it, as the intervention is delivered by the psychiatrist for patients who are randomized to receive it. The RA who recruits and administers assessments to participants is not blinded to condition, except at baseline. The data will be de-identified to maintain blinding during the analysis process.

### Data collection, management, and analysis

#### Data collection

Paper questionnaires are used to collect patient-reported outcomes. The SES is administered to each patient’s primary clinicians. Participants receive a total of $60 in cash for their participation ($20 for the first research interview, $20 for the second research interview, and $10 after each 3- and 6-month follow-up interview). Reimbursement is left in a sealed envelope at the local coordinated specialty care office or sent by mail, as preferred by the participant.

#### Data management

The RA flags any missing answers or comments that suggest a problem with the interview or recruitment process in order to discuss them with the principal investigator (PI; YZI). The RA is responsible for data entry into Microsoft Access, an information management tool that stores information for reference, reporting, and analysis, and IBM SPSS Statistics Version 24. Data quality audits are conducted by the PI and the study team.

#### Analysis plan

Data analysis will be conducted using SPSS. To evaluate the potential effectiveness of the intervention versus treatment as usual on SDM, medication adherence, and service engagement, at the conclusion of an appointment we aim to use independent-samples *t* tests at T_1_ (post-visit) to compare differences in the SDM and adherence/engagement scales. In case of a small sample size, we will use a non-parametric Wilcoxon-Mann-Whitney test [75] or descriptive statistics. To evaluate the effect of the intervention on adherence to decision made, engagement, and knowledge variables over time, we aim to use multivariate analysis of variance (MANOVA). If the sample size is small, we will instead use a non-parametric Friedman test, applying it for each dependent variable separately. We will use descriptive statistics and qualitative information from an implementation report to describe the feasibility of intervention delivery and study procedures. To identify patient-level and intervention-level factors associated with the potential effectiveness of the intervention, we will use chi-square tests and Pearson correlation coefficients between patient characteristics (e.g., age, gender, education level, and diagnosis), personality factors (i.e., attachment style, apathy), clinical relationships (i.e., working alliance, trust), cognitive functioning, and self-stigma.

### Data monitoring

#### Data monitoring and auditing

The PI and RA monitor data internally. The team meets weekly in person or by phone/video to ensure that the project proceeds as intended, per protocol. Participant enrollment rate is tracked weekly, and the study staff complete all items required by the IRB regarding data monitoring. The RA controls the randomization and data storage for the study under supervision of the PI and the research team. Limited data are kept on all individuals who dropped out until the point of leaving the study. Data include age, gender, intervention assignment, and all elements in the eligibility screener. This information will be used to examine non-response bias.

#### Adverse events

There are minimal risks to participating individuals; the main risks are the time and effort involved in completing the interviews. The RA notifies the investigators of any adverse events at regularly scheduled meetings. Study staff keep records of any feedback, questions, concerns, and/or complaints received and address them as needed. The RA has been trained to address adverse events in accordance with the IRB protocol.

### Patient and public involvement

Patients, family members, clinicians, and administrative staff were involved in the development of the intervention [28, 48]. Because the SDM Antipsychotic Medication DA is publicly available, the current study’s questions were informed by the PI (YZI) and potential end-user inquiries about the evidence for the effectiveness of the DA in a prior research [28]. This included questions regarding an interest in how the DA might influence patients’ treatment decisions and engagement in the coordinated specialty care program. The study was also approved by a City IRB committee that includes patient and public representatives in addition to the university IRB (see the “Ethics and dissemination” section). Participants in the City IRB reviewed the intervention, interviews, and research procedures, including how we would contact study participants.

### Limitations

There are some potential limitations to note in the study. First, study staff are not blinded to the intervention, as they are responsible for delivering it (psychiatrists) and for randomization (RA). Although no explicit information is given about the DA to the psychiatrists, there may still be a risk of cross-information contamination between the control and intervention groups given that the same psychiatrist introducing patients to either group may accidentally provide control patients some information about the DA. We will take this limitation in consideration when analyzing the data, especially in case of a nonsignificant trend in a small sample. The preliminary pilot and feasibility results will help us develop a follow-up study. Second, we expect a number of post-randomization exclusions due to participants not showing up for their appointment and a modest amount of attrition over the course of the study. We have implemented standard protocols to maximize response rates that include cash reimbursement at the end of each follow-up and several attempts to contact unresponsive participants. Money provision to research participants is an acceptable approach when conducting research in the U.S. [76, 77], especially with disadvantaged populations who may benefit more from cash. However, the fact that participants know upfront that they will be given cash for their participation may affect recruitment and retention and bias the sample slightly. Third, the follow-up period for SDM variables (post-visit) of 3- and 6-month follow-ups to assess adherence and engagement to capture the impacts of the DA on these variables is exploratory and may not be suitable. Last, because it is a pilot study with the purpose of gathering data to inform future studies and practice, we intentionally did not provide specific guidelines for the use of the SDM Antipsychotic Medication DA and left this up to the psychiatrist’s discretion.

## Ethics and dissemination

### Ethics approval and consent to participate

#### Protocol version

This study protocol was approved by the Temple University IRB on 5 February 2018 and the City of Philadelphia Department of Public Health IRB on 8 December 2017. This manuscript details the protocol on the latest version approved on 12 June 2020 by the Temple University IRB and on 10 December 2019 by the City of Philadelphia IRB.

#### Protocol amendments to the IRB

All changes to the study protocol were reviewed by the IRB. The participating providers and co-investigators were sent regular emails with updates on the study recruitment timeline and any major protocol changes during the enrollment period.

#### Study participant consent

Our process for ensuring that all study participants provide fully *informed* consent involves the following steps. (1) The first line of screening is through contact between research staff and the potential participants (see the “Recruitment strategies, status, and trial dates” section). The research staff provides a brief overview of the study and makes an initial assessment of the participants’ ability to understand and recall the following issues: what participants are asked to do, data collection procedures, and nature of confidentiality. (2) An interview is scheduled with those who are able to recall the information provided and demonstrate an understanding of all areas. Those who have problems with the initial assessment are asked whether they can be contacted later to discuss the project. A second assessment is conducted with the research staff to assess recall from the previous conversation. Perfect recall is not expected. Poorly recalled information is targeted during the review of the informed consent form and study procedures. Research staff spend up to an hour with potential participants reviewing the consent forms. All forms are read aloud with potential participants, and they are again asked to recall information about the study. Prompts are given to facilitate recall if needed. If potential participants are able to recall pertinent information about the study without prompts or with minimal prompting, they are viewed as being able to give informed consent. All consent forms are maintained in the case file along with all identifying information in a locked cabinet that is kept in a secure office.

#### Confidentiality

Special efforts are made to protect the privacy of subjects. All personal identifying information (PII), such as names, addresses, phone numbers, and email addresses, are kept in a secure Access database. Any paper that includes PII is kept in a locked cabinet in a locked office at Temple University. Data management for the study is done through Access. De-identified data are entered into SPSS. All paper surveys and electronic surveys include a patient study ID number and no PII. The Access database that links the study ID number to patient name and contact information is kept separately on a password-protected server.

#### Dissemination plan

To promote dissemination and implementation of the study findings to potential end-users, patients and clinicians, and the scientific community, we will include publication of manuscripts in leading scientific peer-reviewed journals, presentations at scientific meetings and patient organizations in the local, national, and international levels.

#### Process evaluation

A process evaluation was designed to help understand how and why the intervention works. The RA gathers data via the implementation report (completed by the psychiatrist) and patient evaluation form to collect information about any process that might influence study outcomes. These reports include questions about the delivery and receipt of the intervention, treatment recommendation, satisfaction and perception of the participant’s preferred treatment.

## Discussion

This study protocol outlines the approach for a randomized controlled trial evaluating the feasibility and preliminary effect of a novel antipsychotic medication SDM intervention versus treatment as usual on SDM, adherence to treatment decision, and service engagement in FEP care. Understanding how to deliver an SDM Antipsychotic Medication DA as part of a medication management appointment in a coordinated specialty care program for FEP and the potential contribution of SDM compared with usual care in psychosis is critical. The study will help answer several key questions new to SDM research, including the contribution of personality and clinical relationships to SDM in mental health and in psychosis in particular. By contributing evidence on the value of patient and provider decision support strategies, we are eager to offer insights on promoting person-centered care that is believed to be critical in facilitating patient engagement. This fits with recent trends in healthcare policy that emphasize increasing patient involvement in many aspects of care. The results of this study will provide critical evidence for mental health providers, patients, and family members, who are often tasked with making decisions about ongoing medication management.

## Data Availability

Not applicable.

## Abbreviations

FEP: First episode psychosis
DA: Decision aid
SDM: Shared decision making
IRB: Institutional review board
RA: Research assistant
SPIRIT: The Standard Protocol Items: Recommendations for Interventional Trials
CONSORT: The Consolidated Standards of Reporting Trials
RAISE: Recovery After an Initial Schizophrenia Episode
NIMH: National Institute of Mental Health
DSE: Decision Self-Efficacy
DAS: Decision Attitude Scale
DCS: Decisional Conflict Scale
BARS: Brief Adherence Rating Scale
SURF-M: Service Use and Resource Form for Monthly Items
SES: Service Engagement Scale
ECR-R: Experiences in Close Relationships-Revised
WAI: Working Alliance Inventory
BACS: The Brief Assessment of Cognition in Schizophrenia
ISMI: Internalized Stigma of Mental Illness
